# The Unnecessary Extraction of the Dental Pulp through Cataphoresis

**Published:** 1900-06-15

**Authors:** E. A. Bogue

**Affiliations:** New York, N. Y.


### THE UNNECESSARY EXTRACTION OF THE DENTAL PULP THROUGH CATAPHORESIS.

BY E. A. BOGUE, M.D., D.D.S., NEW YORK, N. Y.

Abstract from a paper read before the American Dental Club of Paris, October 7, 1899.

Much is being written of late regarding the removal of pulps through cataphoresis, without discriminating at all as to the teeth from which they are removable surgically, or by immediate instrumentation, which is a process that calls for careful consideration before it is employed. The English journals are already speaking of the danger line, and some of our more advanced American journals seem to be appreciating the fact that the galvanic current may, under certain conditions, do more harm than good. The writer read more than one year and a half ago of the application of the galvanic current to a tooth in which arsenic had previously been placed, with results that may be better imagined than described.

About the time that it was proved possible to render the tooth and its pulp insensible by cataphoresis, a certain gentleman advocated before one of the New Jersey dental associations the extirpation of the pulp for what were considered by some of his hearers very slight reasons, or no reasons at all. This gentleman stated that the office of the pulp was fulfilled when adult life was reached, and that its further presence in the tooth was, in his estimation, a matter of very little consequence. He proceeded, therefore, to advocate its extirpation in many cases where the more conservative operators would have carefully preserved it. So far as I know, no public reply has, up to this time, been made to this paper, and, as nothing has been said against his view, quite a number of operators may have been led to follow these unsound and unwise teachings. As so-called remedies generally appear for any great evil, about this time began to appear and be advocated numerous antiseptic agents, to be used as prophylactics against the putrefaction and decomposition of those remnants of the dental pulp that can not be removed by any process, and those systems were very soon extended to embrace the whole, or the bulk, of the dental pulp, which, it was stated, could be mummified so as to give no further trouble.

Within about a year a case has been brought to my notice in which an effort was made to relieve pain in a nearly exposed pulp in a lower molar by removing it surgically by the aid of cataphoresis. An unmarried lady of good parentage, and no malign tendencies, had been for over twenty years in the hands of the same dentist. During that time she had never suffered from toothache. In the spring of 1898 a right lower molar was slightly broken, and the lady consulted a practitioner in a neighboring city, who, not recognizing the peculiarly delicate physique of the lady, repaired the break with a filling in the ordinary way. The operation itself was exceedingly painful, and the pain experienced during the return journey to New York was such that the lady telegraphed this gentleman, and he came several hours' journey to see her. He removed the filling at the lady's house, and, making an application of carbolic acid, instructed the patient to go to a colleague near by and have the cotton removed if the pain did not subside. As the pain continued during the next day, she went, and, being informed that the gentleman had dismissed a patient expressly to see her, she took his chair and was subjected to a cataphoric application of cocain to this lower molar. The pulp was rendered temporarily insensible, of course, and the practitioner undertook to remove it. So much as he could reach through the small openings that he made was probably removed, but when the effect of the cocain had passed it may readily be imagined that the remnants of torn and lacerated pulp gave no less pain than the exposed dentin had given, for the pulp had not been exposed at all until after the cataphoric application of cocain.

Upon applying to the same gentleman a second time for relief, another filling was removed from another adjoining lower molar. Cocain was driven into this tooth with the electric current, and an attempt was made to remove the pulp from this tooth also, which, of course, could not have been successful, as no instrument made by human hands can reach the entire canal of certain lower molar roots; nor can instruments reach the web-like portion of pulp-tissue that often in flat-rooted teeth spreads from one canal to the other, like the flattened web of a duck's foot.

Again, under the benumbing effect of the cocain, the patient returned home. When the action of the cocain had passed the pain that follows a lacerated pulp returned, and, of course, inflammation of that organ must have supervened.

At this stage the patient was taken to an oral surgeon, who made an examination by transillumination, and *thought* he located a dark shadow in the antrum, which, accordingly, he opened through the nasal passage on that side, evacuating something more than half a teaspoonful of what appeared to be pus or muco-purulent matter, though no accurate knowledge can be had of the matter, as no careful examination was made of it. Under the influence of this fresh pain the patient tried to rest content for a few days, but the generally neurotic condition was such that she went once again to visit the oral surgeon, and this time a perfectly sound and healthful upper bicuspid was extracted and a fresh perforation was made into the antrum.

In view of the artificial opening which had been made through the nares and the necessary admission of foreign substances, which would naturally cause the formation of pus, there was evacuated this time considerable pus, and some care was taken and some pain given in thoroughly washing out the antrum and getting it once more into an aseptic condition.

The pain still continued, and the next move was an opening (the father says) into the submaxillary gland, but at any rate down to the carotid artery, evacuating a small quantity of blood from beneath the surrounding fascia, for interference with the circulation in a painful region has been known to relieve or prevent pain in the affected part. Trousseau, of France, and Nussbaum, of Germany, have ligated arteries, successfully controlling pain by the process. In this case the pain seemed to subside for several days; not disappearing entirely, but being less severe. The subsidence was not for long, however, for very soon these two lower molar teeth began to be so very painful that, worn out by this long suffering, which had caused the loss of fourteen pounds in weight, the lady consented to the extraction of one of the lower molars, not the one from which the first pulp was supposed to have been removed, however. The operation was successfully performed, but little, if any, relief from pain was experienced.

Two other professional gentlemen were now consulted; the one an electrical specialist giving his attention largely to cataphoresis, the other a dentist giving some attention to oral surgery, and the treatment was to remove the pulp, or as much of it as possible, from the upper first molar, cataphoresis again being resorted to for this purpose. The pain still continuing below, an application of cocain every forty-eight hours or thereabouts was adopted as a palliative, in hopes of procuring rest for the sufferer. (No one seems to have thought of removing the guttapercha filling put in by the first dentist who undertook to remove the first pulp from the lower molar by cataphoric help).

At this stage the one who had charge of the lady's teeth during twenty years was consulted, "being asked if he knew anything about nerves independently of teeth," but he declined to hazard an opinion under the then existing conditions, not feeling himself competent to advise where so many others had failed to give relief, especially as it seemed evident that surgical interference would become necessary. At this time the pain was so intense that the screams of the patient at night were uncontrollable, and the constant continuance of the pain seemed to call for a surgical operation. Accordingly, a few days thereafter the jaw was opened externally and the inferior dental nerve was divided, and I am informed a portion was exsected.

After the wound of this last serious operation was well healed attention was again called to the immediate seat of the first difficulty—namely, the right second lower molar, upon which the cataphoric current had first been directed, and from which the pulp was supposed to have been taken painlessly. A dentist in the country was requested to examine the case. His first step was to remove the gutta-percha filling inserted by the one who was said to have removed the pulp nearly five months previously. A little probing with so-called nerve instruments revealed the presence of living pulp in this tooth, which in turn was extracted. This tooth has been in my possession for a number of weeks. The drill hole into the posterior root is round and scarcely larger than the orifice of *one* of the canals that it was desired to clear out. The anterior root had been cut off before I saw the tooth. When I first saw it the pulp was still in it. This was several months after the beginning of the trouble, and during all that time this lacerated pulp continued living, and continued to behave like an exposed pulp, until it was discovered by the country practitioner who removed the tooth.

The points to which I especially wish to call your attention are, first, that complete insensibility of the dentin in the small operations that we are called upon day by day to perform is not desirable. Unless a man is far more expert in minute dental anatomy than the majority of practitioners can be, he needs the guidance given him by a slight sensibility when he is excavating. All the obtunding that is required can generally be procured by the use of pure carbolic acid and cocain, followed by a crystal of zinc chlorid, if that can be allowed to remain ten minutes. For all ordinary cases cocain or eucain beta in ether, as it is now furnished, or pure carbolic acid, will be found amply sufficient.

Again, we are not authorized needlessly to destroy, and the destruction of a pulp or the extraction of a tooth needlessly is an acknowledgment *per se* of our inability to save; and unless the good to be obtained by either of these destructive processes, or any other, for that matter, is far greater than the loss to which the patient is to be subjected, we have no right to do either.—*Dental Cosmos.*

